# Pelleting of a Total Mixed Ration Affects Growth Performance of Fattening Lambs

**DOI:** 10.3389/fvets.2021.629016

**Published:** 2021-02-12

**Authors:** Bo Li, Xuezhao Sun, Qin Huo, Guiguo Zhang, Tingting Wu, Peihua You, Yuhua He, Wannian Tian, Rongquan Li, Changsheng Li, Jianping Li, Chunqing Wang, Baijun Song

**Affiliations:** ^1^The Innovation Centre of Ruminant Precision Nutrition and Smart and Ecological Farming, Jilin Agricultural Science and Technology University, Jilin City, China; ^2^Jilin Inter-Regional Cooperation Centre for the Scientific and Technological Innovation of Ruminant Precision Nutrition and Smart and Ecological Farming, Jilin City, China; ^3^College of Animal Science and Technology, Shandong Agricultural University, Taian City, China; ^4^Portal Agri-Industries Co., Ltd., Nanjing City, China

**Keywords:** fattening lamb, growth performance, pelleting, total mixed ration, physical form of feed

## Abstract

Feeding pelleted total mixed rations (TMR) instead of traditional loose concentrate plus forage to fattening lambs is an emerging practice. This study aimed to determine the effects of feeding pelleted TMR to fattening lambs on feed intake behaviour, growth performance, feed digestion, rumen fermentation characteristics, rumen microbial community, serum parameters, slaughter performance, meat quality, and the economic outcome. Two physical forms (pelleted vs. un-pelleted) of TMR composed of the same ingredients with the same particle sizes were compared in three animal experiments. Feed intake and average daily gain were higher when the TMR was pelleted, but apparent total tract digestibility of nutrients (organic matter, crude protein, neutral detergent fibre, acid detergent fibre, and ether extract) and serum parameters were not affected and apparent total tract dry matter digestibility was slightly lower. Feeding pelleted TMR increased total short-chain fatty acid concentration and decreased rumen pH. Rumen microbial community was not affected by the physical form of the TMR at phylum level but changed slightly at genus level. Liveweight at slaughter and hot carcass weight were higher for lambs fed the pelleted compared to the un-pelleted TMR, while dressing percentage and meat quality were not affected. In conclusion, feeding pelleted TMR improves growth performance of fattening lambs mainly due to an increase in feed intake. Feeding pelleted TMR is a feasible strategy for intensive lamb fattening operations.

## Introduction

Since being introduced to ruminant production systems over a half century ago, total mixed rations (TMR) have nowadays become common practice (1–3). Although there are many advantages of TMR, this feeding system also has some limitations (2), particularly feed sorting, which need to be addressed (4). This led to the recent trend to offer partially or completely pelleted TMR to dairy cows and heifers in some regions of the world (5–7). The use of pelleted TMR has also been reported for goat (8, 9) and sheep production (10–13).

Pelleting was introduced to the feed industry in the 1920s and is widely adopted in the poultry and swine industry (14, 15). Ground hay was pelleted in the 1950s and 1960s to improve its feeding value for ruminants (16). However, due to the lack of mobile pelleting presses, high processing cost, and the limited improvement in feeding value, the interest in pelleting forages declined. Improvements in pelleting technology and the recognition of the advantages of pelleting feed for poultry and swine have increased the interest in feed pelleting for ruminants again. At present, forages like lucerne (*Medicago sativa* L.) are sometimes pelleted for use in ruminants (17, 18). Pelleting reduces the volume of the material and therefore cost of transportation (17). In addition, the use of pelleted concentrates is common in ruminant production systems as well, for example, in the dairy industry (19). Supplements used during winter-feeding of sheep and beef cattle outdoors are also commonly pelleted to reduce losses to the environment. However, pelleting of complete diets is still uncommon in most ruminant production systems.

In theory, a pelleted TMR is expected to have advantages over an un-pelleted TMR, particularly in feeding systems in which the diet ingredients are not mixed before feeding but offered separately (20). Due to the elimination of feed sorting and the fact that the feed is thoroughly mixed before pelleting (21), the nutrient intake is more uniform if feed is pelleted (22). This could stabilise the rumen environment and consequently reduce the risk of acute and subacute rumen acidosis. However, the stabilisation of rumen pH could be counteracted by the reduction of the physical effectiveness of fibre due to pelleting. Another positive aspect of pelleting is that the feed is heated during pelleting process, which partially gelatinises starch and can denature proteins and antinutritional substances. This can result in a positive impact on digestibility (23). Pelleting also makes it easier to include some less palatable by-products (24). Furthermore, the production of pelleted TMR in commercial feed plants might result in more professional quality control of the diet ingredients and resulting TMR. In addition, pellets have a higher density compared to un-pelleted feed, which makes it easier and more cost-effective to transport and store pellets. Also, nutrient losses of pellets are lower since they are completely dry and less prone to spoilage and shrink. Feeding pelleted TMR can also reduce on-farm labour cost, as forage handling and feed mixing are not required.

We hypothesise that compared to ground TMR pelleting increases feed intake and digestibility, manipulates rumen microbial community, enhances rumen fermentation and results in improved growth in fattening lambs when the particle size of TMR is the same. The objective of this study was to determine effects of feeding pelleted TMR vs. un-pelleted TMR with the same particle sizes on feed intake behaviour, growth performance, feed digestion, rumen fermentation characteristics and microbial community, serum parameters, slaughter performance, meat quality, and the economics of fattening lambs.

## Materials and Methods

### Experimental Design, Animals, and Management

This study compared two physical forms (pelleted vs. un-pelleted) of TMR composed of the same ingredients with the same nutrient composition in three animal experiments. Experiments 1 and 2 (Exp 1 and Exp 2) were conducted at the Animal Experimental Station of Jilin Agricultural Science and Technology University, Jilin City, Jilin Province, China. Experiment 3 (Exp 3) was conducted on a commercial farm in Tongyu County, Jilin Province, China. Experiment 1 investigated growth performance, digestibility, serum parameters, rumen fermentation parameters and microbial community, feed intake behaviour, slaughter performance, meat quality, and economic benefits. Experiment 2 investigated growth performance, rumen fermentation parameters and microbial community, slaughter performance, meat quality, and economic benefits. Experiment 3 only examined growth performance. In this study, lambs with different liveweights and different sexes were used. The aim of this design was to draw conclusions that apply to different production conditions. All lambs were tested for brucellosis (*Brucella* spp.) using the Rose Bengal plate assay (25), and only brucellosis-negative lambs were included in the experiments.

For Exp 1, 24 three-month-old healthy uncastrated male F2 hybrids of thin-tailed sheep and Northeast fine-wool sheep, the predominant crossbred in Northeast China, with a liveweight of 26.3 ± 3.1 kg were used for the experiment. The lambs were adapted to the housing conditions for 14 days, orally de-wormed with albendazole at a dose of 15 mg per kg liveweight, and shorn. Sixteen healthy lambs were selected for the experiment. The selected lambs were stratified by liveweight, and randomly allocated to one of the two dietary treatments. The lambs were adapted to their designated experimental feed within 3 days by gradually increasing the level of the experimental feed in the diet. Then these lambs were fed their designated experimental diet until the completion of the experiment. The lambs were weighed fortnightly before the morning feeding using an electronic scale with a precision of 0.05 kg (Dahe Electronics Co., Ltd., Wuyi, Zhejiang, China). Average daily gain (ADG) was calculated as the slope of the regression of liveweight against time. After 2 weeks, the lambs were transferred to metabolic crates to determine the apparent total tract digestibility of nutrients. The lambs were adapted to the crates for 6 days and the digestibility measurement lasted for 7 days. During the digestibility period, one lamb of each treatment was sick due to an injured leg and removed from the experiment. On day 28, rumen and blood samples were taken from all lambs. Rumen samples were collected 0 and 3 h after the morning feeding. Blood samples were taken directly before morning feeding. Between day 62 and 66, feed intake behaviour was monitored over 5 consecutive days. On day 67, growth performance measurement was completed and rumen and blood samples were taken. Sampling was carried out following the same protocol as on day 28. On day 68 and 69, three lambs of each treatment were slaughtered each day.

The experimental design and animal management in Exp 2 was the same as in Exp 1. Sixteen 5-month-old uncastrated male lambs with a liveweight of 43.8 ± 4.0 kg after an adaptation period were randomly allocated to one of the two dietary treatments (*n* = 8). Growth performance was measured for 24 days. After the measurement, lambs were fed their designated diet for two additional days. One day after the completion of the growth performance measurement rumen and blood samples were collected following the same protocol as in Exp 1. Then three lambs from each treatment were slaughtered on each day of the next 2 days.

Experiment 3 was conducted with 36 ewe lambs with a liveweight of 25.0 ± 3.0 kg. After a 10-day adaptation period, lambs were stratified by liveweight to have 18 blocks, and two lambs in each bock were randomly allocated to one of the two dietary treatments. The growth performance measurement lasted for 29 days.

### Experimental Diet and Feeding

The experimental diet offered in all three experiments was formulated according to the Chinese Feeding Standard for Lamb Finishing (26). The ingredient and chemical composition of the diet is shown in Table 1. Sorghum husks and maize grain were dry-rolled, while the other ingredients, including maize germ meal, sunflower seed meal, peanut shells, rice hulls, cottonseed meal, and barley malt rootlets were passed through a combined 4 and 6 mm screen (half of the screen: 4 mm diameter holes, other half of the screen: 6 mm holes). All ingredients were thoroughly mixed. Half of resulting TMR was pelleted, and the other half was kept un-pelleted as loose mash. The pelleting conditions were, conditioning at 85°C for 45 s, pelleting at 90°C, and subsequent forced air cooling. The ring die compression ratio was 1:7. All pellets for the three experiments were produced in a single batch using the same pelleting press (model YPM508E, Jiangsu Yongli Machinery Co., Ltd., Liyang, Jiangsu, China) at the Chifeng subsidiary Company of Jiangsu Portal Agri-Industries Co., Ltd., China. The pellets were 5 mm in diameter and 8–10 mm in length and stored in waterproof bags in the dark.

**Table 1:** Ingredients and chemical composition of experimental diets.

	**Diet**	
**Item**	**Pelleted**	**Un-pelleted**
Ingredient (kg/t of fresh weight)		
Maize	350	350
Maize germ meal	120	120
Sunflower seed meal	120	120
Peanut shells	113	113
Rice hulls	70	70
Cottonseed meal	30	30
Bentonite	20	20
Barley malt rootlets	100	100
Limestone	14	14
Sorghum husks	10	10
Calcium hydrogen phosphate	7	7
Ground soybeans	20	20
Sodium chloride	6	6
Trace mineral and vitamin premix^a^	20	20
Nutrient content^b^ (g/kg of DM)		
Dry matter (DM) (g/kg of fresh weight)	880	879
Organic matter (OM)	903	901
Crude protein (CP)	158	161
Neutral detergent fibre (NDF)	427	418
Acid detergent fibre (ADF)	174	173
Ether extract (EE)	16	19
Metabolizable energy (MJ/kg of DM)^c^	11.7	11.8

a *Premix per kg contained 200,000 IU vitamin A, 60,000 IU vitamin D_3_, 550 mg vitamin E, 800 mg nicotinamide, 650 mg Cu (as CuSO_4_), 2,800 mg Fe (as FeSO_4_), 900 mg Mn (as MnSO_4_), 16 mg Se (as Na_2_SeO_3_), 3,600 mg Zn (as ZnSO_4_), 20 mg Co (as CoCl_2_), 15 mg (as Ca(IO_3_)_2_), and 15 g lysine. The carrier was composed of glucose, rice bran, zeolite powder, and limestone powder*.

b *The nutrient contents were measured values*.

c *Metabolizable energy was estimated from NRC (27)*.

Lambs were individually fed in metabolic crates during the digestibility measurement period, while they were group fed in pens during the remaining time of the experiments. Lambs were fed equal amounts twice per day (800 and 1600 h). Refusals were collected and quantified daily to continuously adjust the feed allowance. At least 10% refusal was allowed to achieve *ad libitum* feeding. Water was available all the time. Animal behaviour and health were monitored, and weather, air temperature and humidity were recorded daily. Feed troughs, pens and metabolic crates were cleaned before the morning feeding.

### Apparent Total Tract Digestibility Measurements

Total faeces collection was used to determine the apparent total tract digestibility of nutrients (28). One day before the start of the total collection, the lambs were fitted with the collection harnesses to allow adaptation to the equipment. Feed provided, refusal, and faeces were quantified daily during the 7-day sample collection period. Feed samples (50 g) were collected each day and pooled for each dietary treatment. Refusals were kept, pooled for each animal and subsampled at the end of the experiment. One percent of daily faeces was acidified with 10% H_2_SO_4_ at a ratio of 1:10 (w/w) and another subsample of 10% of the daily faeces was subsampled and not acidified. The daily samples were stored at −20°C and pooled and subsampled over each animal at the end of the experiment.

### Rumen and Blood Sampling

Approximately 5 ml of blood was collected from the jugular vein into coagulation promoting tubes with separating gel (Sanli Industrial Co., Ltd., Huizhou, China). Rumen contents were collected using an oesophagal tube (12). The pH of the rumen samples was measured immediately after sampling using a pH meter (LICHEN pH-100A, Shanghai Lichen Scientific Laboratory Instrument Ltd., Shanghai, China). The samples were kept on ice and brought to the laboratory within 30 min and subsampled into 2-ml cryogenic vials (Corning Inc., New York, USA). Subsamples were stored at −20°C for short-chain fatty acid (SCFA) and ammonia measurements and at −80°C for the analysis of the rumen microbial community (3 h after morning feeding samples only).

### Feed Intake Behaviour

Feed intake behaviour was recorded for five consecutive days in Exp 1 using a digital video camera (Model C3W 720P; Hangzhou Hikvision Digital Technology Co., Ltd., Hangzhou, China). The time distribution and duration of feed intake were observed and counted using the playback function of the system.

### Slaughter, Carcass Performance, and Meat Quality

Lambs were fasted for 24 h, weighed to determine their liveweight (LW), and slaughtered by exsanguination. Hot carcass weight (HCW) was recorded immediately after slaughter with suet and kidneys included. Dressing percentage was calculated as HCW divided by LW × 100. The loin eye area was estimated according to Luo et al. (29) based on the loin eye width and height (eye muscle area (mm^2^) = loin eye width (mm) × loin eye height (mm) × 0.7). The width and height in the equation were measured from the cut surface of *longissimus dorsi* muscle between the 12th and 13th ribs using a digital Vernier calliper with a precision of 0.01 mm (DL91150; Deli Group Ltd, Ningbo, Zhejiang, China). Organs were immediately weighed after slaughter using an electronic scale with a precision of 1 g (DH-2012; Diheng Electronic Co., Ltd, Shenzhen, Guangdong, China). Organ indexes were calculated as organ weights divided by LW. After slaughter, about 300 g of the *longissimus dorsi* muscle was taken and placed in a self-sealed bag and placed on ice in a Styrofoam box. Samples were refrigerated at 4°C until analysed. Meat colour, brightness, marbling, pH value, shear force, and water-holding capacity were measured after 36 h.

After hanging for 36 h at 4°C, the meat samples were diced to a height of 30 mm and a thickness of 30 mm. Meat pH value was measured by inserting a pH probe (model pH-STAR; Matthäus GmbH, Poettmes, Germany) into the meat (depth: 15 mm). Meat colour and marbling were scored using the US NPPC meat quality scoring card. Meat brightness was measured using the carcass colour monitor (model OPTO-STAR; Matthäus GmbH, Poettmes, Germany). Shear force was measured using a digital muscle tenderness meter (model C-LM 3B; the Engineering College of Northeast Agricultural University, Harbin, China) in the method described by Santos-Silva et al. (30). Water-holding capacity was measured in the filter paper press Grau and Hamm method (31) using a meat water-holding capacity tester (Model RH-1000; Guangzhou Runhu Instruments Co., Ltd., Guangzhou, China) and expressed as percentage of remaining muscle weight relative to the original weight before applying external force.

### Laboratory Analyses

Feed, refusal, and faeces samples were dried at 65°C for at least 48 h to achieve constant weight, ground to pass a 1 mm screen using a Wiley mill (Arthur H Thomas, Philadelphia, PA, USA), and determined for the concentrations of dry matter [DM; (32), ash (33), crude protein [CP; method no. 968.06; (34)], fibre, and ether extract [EE; (35)]. The concentration of CP in faeces was determined using acidified samples, while other nutrients were analysed from unacidified samples. Organic matter (OM) was calculated as 1,000 minus ash content (g/kg). The fibre contents were determined consecutively as ash-free neutral detergent fibre (aNDFom) with heat-stable α-amylase and sodium sulfite, and ash-free acid detergent fibre (ADFom) according to van Soest et al. (36).

Serum was harvested by centrifuging the blood sample at 1,000 × *g* for 5 min (Model TDL-80-2B; Anting Scientific Instrument Factory, Shanghai, China) and analysed using an automatic biochemical analyser (Model 7160; Hitachi Ltd., Tokyo, Japan) with reagent kits from Mairui Biomedical Electronics Co., Ltd. (Shenzhen, China). Following blood parameters were analysed: total protein (TP), albumin (ALB), blood urea nitrogen (BUN), creatinine, glucose, triglyceride (TG), cholesterol, high-density lipoprotein cholesterol (HDL), low-density lipoprotein cholesterol (LDL), α-amylase, lipase, alanine transaminase (ALT), aspartate transaminase (AST), and alkaline phosphatase (ALP). Globulin (GLB) was calculated as TP minus ALB.

Rumen samples for the determination of SCFA and ammonia concentrations were thawed, centrifuged at 4°C in 8,000 × *g* for 10 min, filtered, and analysed using the method described by Huo et al. (12). The identification and quantification of SCFA were performed with an FFAP 30 m × 3 mm × 0.25 μm capillary polar column using a gas chromatograph system (model GC9790; Fuli Instruments Ltd., Wenling, Zhenjiang, China) fitted with a flame ionisation detector, while ammonia was quantified using the Indigo phenol blue-spectrophotometry method (37) modified by Feng and Gao (38).

The rumen bacterial community profile was characterised according to Huo et al. (12). Briefly, total genome DNA was extracted using the E.Z.N.A.® Soil DNA Kit (Omega Bio-tek, Norcross, GA, U.S.A.), and the 16S rRNA genes were amplified using hypervariable V3-V4 region PCR primers (341F: 5-CCTAYGGGRBGCASCAG-3; 806R: 5-GGACTACNNGGGTATCTAAT-3). Sequencing libraries were generated using NEB Next®Ultra™DNA Library Prep Kit for Illumina (New England Biolabs, Ipswich, MA, USA), and sequenced on an Illumina MiSeq platform (Illumina, Inc., San Diego, CA, USA; 250 bp/300 bp paired-end). Paired-end reads from the original DNA fragments were merged using FLASH (39). Sequences analysis were performed by UPARSE software package using the UPARSE-OTU and UPARSE-OTU ref algorithms (40). Sequences with ≥97% similarity were assigned to the same operational taxonomic units (OTUs), and the taxonomic assignment of each OTU was performed using the RDP Classifier according to the database Silva (www.arb-silva.de) updated for ruminal bacteria (41). The Mothur software package v.1.21.1 (42) was used to estimate bioinformatics parameters.

### Economic Benefits

The economic analysis was performed by deducting costs of production from the income of selling the fattened lambs. The costs of production were estimated using the market prices during the time the experiments were conducted. Chinese yuan was converted to US dollars at a ratio of 650 Chinese yuan to 100 US dollars. The purchasing price of lambs was US$4.31/kg in Exp 1 and US$3.69/kg in Exp 2, while the selling price was US$3.85/kg. The fixed cost for labour, medical prevention and treatment, and water and electricity supply were respectively US$3.08, 0.46, and 0.62 per lamb in Exp 1 and US$1.08, 0.31, and 0.31 in Exp 2. Estimates were calculated based on average costs of sheep producers in the region. The feed price was US$0.35 per kg for the pelleted and US$0.34 per kg of un-pelleted TMR.

### Statistical Analysis

Liveweight, ADG, feed intake behaviour, apparent total tract digestibility, blood biochemical parameters, slaughter performance, and meat quality were analysed separately for each experiment with an one-way ANOVA using GenStat 19th edition (VSN International, Hemel Hempstead, UK, 2017) (43). As lambs were group fed in pens, the feed to gain ratio could not be statistically analysed for each experiment. Instead, the feed to gain ratios from Exp 1 and Exp 2 were analysed together with experiment as block using a one-way ANOVA. Rumen bacterial community data were first analysed separately for Exp 1 and Exp 2, and similar results were obtained. These data from the two experiments were analysed together with experiment and feed physical form as two experimental factors using the two-way ANOVA. Since the interactions between these two factors and the differences between the two experiments were not significant, the data were analysed with feed physical form as the experimental factor only using the one-way ANOVA. The significance of difference was declared at *P* < 0.05, tendency at 0.05 < *P* < 0.10.

## Results

### Growth Performance and Apparent Total Tract Digestibility

Lambs ate 34% and 15% more (*P* < 0.001) in Exp 1 and Exp 2, respectively, when pelleted feed was provided compared to un-pelleted feed (Table 2). The ADG was 38% higher (*P* = 0.003) for lambs fed pelleted feed than those fed un-pelleted feed in Exp 1 and 19% higher in Exp 2, although the difference was not statistically significant. In Exp 3, lambs consuming pelleted feed also had 21% higher ADG than those consuming un-pelleted feed. The ratio of feed to liveweight gain did not differ between the two physical forms of feed (*P* = 0.411) when the data from Exp 1 and Exp 2 were analysed together.

**Table 2:** Effects of dietary physical form (pelleted vs. un-pelleted) on growth performance of fattening lambs.

				**Diet**			
**Exp**	**Site**	**Sex**	**Item**	**Pelleted**	**Un-pelleted**	**SEM**	***P*-value**
1	Jilin	Male	Number of lambs	7	7		
			Length of the experiment (d)	67	67		
			Initial liveweight (kg)	24.6	24.7	0.71	0.891
			Liveweight on d 28 (kg)	31.4	29.5	0.93	0.176
			Final liveweight on d 67 (kg)	44.6	38.9	1.14	0.004
			ADG^a^ from d 0 to 28 (g/d)	230	161	16.4	0.011
			ADG from d 28 to 67 (g/d)	334	232	20.4	0.004
			ADG (g/d)	296	214	15.4	0.003
			Daily feed intake (kg DM)	1.49	1.12	0.037	<0.001
			Feed/Gain	5.03	5.20		
2	Jilin	Male	Number of lambs	8	8		
			Length of the experiment (d)	24	24		
			Initial liveweight (kg)	43.6	44.1	1.47	0.806
			Final liveweight (kg)	48.9	48.1	1.51	0.716
			ADG (g/d)	198	167	35.7	0.547
			Daily feed intake (kg DM)	1.78	1.55	0.039	<0.001
			Feed/Gain	7.98	9.19		
3	Tongyu	Female	Number of lambs	18	18		
			Length of the experiment (d)	29	29		
			Initial liveweight (kg)	24.9	23.9	0.79	0.363
			Final liveweight (kg)	30.9	28.8	0.86	0.103
			ADG (g/d)	207	170	12.2	0.055

a *ADG, average daily gain*.

During the digestibility measurements in Exp 1, DM intake was 18% higher for lambs fed pelleted feed than those fed un-pelleted feed (*P* < 0.001; Table 3). The apparent total tract DM digestibility of pelleted feed was 3.1% lower (*P* = 0.038) compared to un-pelleted feed. The apparent total tract digestibility of OM, OM, CP, aNDFom, ADFom, and EE were similar for the two feed physical forms.

**Table 3:** Effects of feeding pelleted vs. un-pelleted feed on apparent total tract nutrient digestibility of fattening lambs (*n* = 7 per treatment).

	**Diet**			
**Index**	**Pelleted**	**Un-pelleted**	**SEM**	***P*-value**
Dry matter intake (DMI, g)	1,373	1161	32.7	0.001
Digestibility (%)				
Dry matter (DM)	61.6	64.7	0.92	0.038
Organic matter (OM)	66.0	66.1	0.93	0.758
Crude protein (CP)	70.2	72.0	1.00	0.227
Neutral detergent fibre (aNDFom)	50.2	50.5	1.29	0.890
Acid detergent fibre (ADFom)	28.4	30.4	1.96	0.479
Ether extract (EE)	92.5	94.3	1.62	0.446

### Rumen Fermentation and Microbial Community

Lambs fed pelleted feed tended to have lower rumen pH both before (*P* = 0.051 in Exp 1, *P* = 0.091 in Exp 2) and after the morning feeding (*P* < 0.05). The drop in pH was 0.25–0.31 units 3 h after the morning feeding compared to un-pelleted feed (Table 4). There were no differences in rumen ammonia concentrations between the two dietary treatments in Exp 1, but in Exp 2, ammonia concentrations were higher (*P* = 0.026) before morning feeding and tended to be higher (*P* = 0.089) 3 h after morning feeding for lambs fed pelleted feed compared to un-pelleted feed. The concentration of total SCFA in the rumen of pellet-fed lambs was higher (*P* < 0.031) than that in the rumen of lambs fed un-pelleted feed before morning feeding and numerically higher (*P* ≤ 0.134) 3 h after morning feeding in Exp 1 and Exp 2. The ratio of acetate to propionate and molar proportions of individual SCFAs did not differ between the two feed physical forms in Exp 1, but the ratio and molar proportion of acetate were higher (*P* ≤ 0.029) for lambs fed pelleted feed than those fed un-pelleted feed both before and after feeding in Exp 2. However, the molar proportion of propionate was similar between the two treatments.

**Table 4:** Effects of feeding pelleted vs. un-pelleted feed on rumen fermentation parameters before and 3 h after morning feeding.

		**Before morning feeding**				**3 h after morning feeding**			
**Exp**	**Item**	**Pelleted**	**Un-pelleted**	**SEM**	***P-*value**	**Pelleted**	**Un-pelleted**	**SEM**	***P*-value**
1	Number of lambs sampled	7	7			7	7		
	pH	6.92	7.02	0.042	0.051	6.04	6.36	0.062	0.029
	NH_3_-N (mg/dl)	8.7	8.2	0.77	0.638	17.0	18.4	1.40	0.468
	Total SCFA^a^ (mM)	39.9	28.1	3.33	0.031	71.1	59.9	4.63	0.116
	Acetate/propionate	2.76	3.30	0.394	0.349	1.92	2.30	0.289	0.378
	SCFA (%)								
	Acetate	56.5	58.9	1.85	0.387	53.5	56.8	1.75	0.212
	Propionate	24.4	18.7	2.95	0.199	32.0	25.9	3.23	0.209
	Butyrate	14.1	16.6	1.38	0.222	13.0	15.4	1.87	0.371
	*iso*-Butyrate	1.8	2.1	0.22	0.307	0.6	0.8	0.11	0.164
	*iso*-Valerate	3.2	3.7	0.41	0.411	1.0	1.1	0.20	0.695
2	Number of lambs sampled	8	8			8	8		
	pH	6.89	7.07	0.072	0.091	6.00	6.25	0.055	0.005
	NH_3_-N (mg/dl)	16.9	12.0	1.47	0.026	19.0	12.7	2.42	0.089
	Total SCFA, (mM)	41.5	27.1	4.11	0.029	59.5	47.8	5.08	0.134
	Acetate/propionate	3.18	2.25	0.203	0.012	2.19	1.69	0.133	0.029
	SCFA (%)								
	Acetate	61.6	55.9	1.49	0.017	58.1	55.2	1.32	0.029
	Propionate	15.8	20.3	1.83	0.101	22.9	27.7	2.74	0.272
	Butyrate	18.9	15.9	1.40	0.160	17.7	14.9	1.83	0.318
	*iso*-Butyrate	1.3	3.6	0.80	0.057	0.5	0.9	0.08	0.018
	*iso*-Valerate	2.5	4.3	0.56	0.042	0.8	1.4	0.13	0.008

a *SCFA, short-chain fatty acids*.

In Exp 2, pH was measured in the digestive tract at slaughter. The pH in the rumen, reticulum, and omasum of lambs fed pelleted feed was lower, compared to lambs offered un-pelleted feed (Supplementary Table 1).

**Figure 1:**
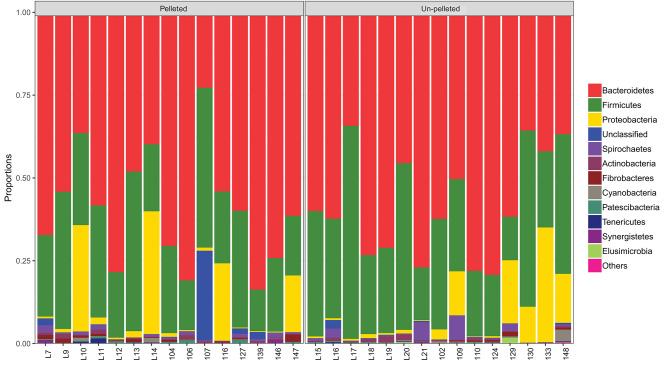
Distribution of the most dominant phyla in the rumen of fattening lambs fed pelleted vs. un-pelleted feed. Phyla with relative abundance of <2% were combined as other.

In the analysis of rumen microbial community, effective reads per sample and the number of OTUs were not affected by the physical form of the feed, averaging 42,802 and 960 respectively, in Exp 1 and Exp 2. At phylum level, Bacteroidetes were most abundant, accounting for over 50% of total bacteria, Firmicutes (28–32%) were second, and Proteobacteria (about 4%) were third most abundant (Figure 1 and Supplementary Table 2). The three predominant phyla accounted for over 95% of all phyla. Pelleting did not affect the abundance of phyla.

At genus level (Table 5), pelleting tended to decrease the abundance of *Prevotellaceae UCG-001* from 3.1 to 0.9% (*P* = 0.070), *Succiniclasticum* from 1.6 to 0.4% (*P* = 0.080), *Prevotellaceae_uncultured* from 0.3 to 0.1% (*P* = 0.077), and *Erysipelotrichaceae UCG-002* from 0.2 to 0.0% (*P* = 0.068), but increase the abundance of *Fibrobacter* from 0.3 to 0.6% (*P* = 0.076), *Candidatus Saccharimonas* from 0.1 to 0.3% (*P* = 0.058), and *Clostridiales vadinBB60 group_norank* from 0.1 to 0.3% (*P* = 0.086) in Exp 1 and Exp 2.

**Table 5:** Effects of feeding pelleted vs. un-pelleted feed on the relative abundance (%) of ruminal bacteria at the level of genus in fattening lambs, samples collected before the morning feeding (*n* = 15 per treatment).

	**Diet**			
**Genus^a^**	**Pelleted**	**Un-pelleted**	**SEM**	***P*-value**
*Prevotella 1*	33.4	27.2	5.59	0.440
*Prevotella 7*	11.5	15.4	4.31	0.520
*Rikenellaceae* RC9 gut group	5.7	3.5	1.37	0.277
*Succinivibrio*	4.2	4.3	2.05	0.974
*Ruminococcus* 2	3.2	4.8	2.26	0.634
*Succinivibrionaceae UCG-001*	3.3	1.9	1.63	0.541
*Selenomonas 1*	1.5	2.9	1.74	0.593
*Lachnospiraceae NK3A20 group*	2.0	2.2	0.56	0.864
*Prevotellaceae UCG-001*	0.9	3.1	0.79	0.070
*Muribaculaceae_norank*	1.6	2.3	0.68	0.502
*Prevotella* 9	1.5	1.7	1.07	0.904
*Unclassified*	2.3	0.3	1.26	0.285
*F082_norank*	1.6	1.0	0.54	0.414
*Dialister*	1.5	1.0	0.59	0.518
*Christensenellaceae R-7 group*	1.5	0.9	0.33	0.244
*Ruminococcaceae UCG-014*	1.0	1.4	0.32	0.331
*Veillonellaceae_uncultured*	1.0	1.3	0.66	0.782
*Lachnospiraceae ND3007 group*	1.0	1.2	0.60	0.771
*Treponema 2*	0.8	1.4	0.42	0.308
*Shuttleworthia*	1.1	1.1	0.57	0.981
*Syntrophococcus*	0.6	1.5	0.61	0.290
*[Eubacterium] coprostanoligenes group*	1.2	0.8	0.25	0.289
*Succiniclasticum*	0.4	1.6	0.47	0.080
*Selenomonas 3*	1.1	0.9	0.74	0.802
*Veillonellaceae UCG-001*	0.5	1.0	0.44	0.448
*Prevotellaceae UCG-003*	0.7	0.8	0.31	0.913
*Alloprevotella*	0.4	1.0	0.36	0.209
*Oribacterium*	0.6	0.8	0.29	0.629
*Saccharofermentans*	0.6	0.7	0.34	0.923
*Ruminococcaceae NK4A214 group*	0.5	0.7	0.15	0.243
*Lachnospiraceae_uncultured*	0.5	0.6	0.30	0.827
*Prevotellaceae_Unclassified*	0.6	0.5	0.18	0.861
*Roseburia*	0.5	0.5	0.30	0.975
*Ruminococcus* 1	0.4	0.5	0.11	0.642
*Fibrobacter*	0.6	0.3	0.13	0.076
*Erysipelotrichaceae UCG-004*	0.5	0.2	0.12	0.138
*CAG-352*	0.0	0.5	0.30	0.307

a *Genera with a relative abundance of <0.5% are not listed*.

### Serum Parameters

Pelleting did not affect any of the serum parameters assayed in Exp 1 (Table 6).

**Table 6:** Effects of feeding pelleted vs. un-pelleted feed on serum parameters of fattening lambs in Exp 1 (*n* = 7 per treatment).

	**Diet**			
**Parameter**	**Pelleted**	**Un-pelleted**	**SEM**	***P*-value**
Protein metabolism				
Total protein (g/L)	75.8	73.2	1.76	0.303
Albumin (g/L)	27.5	26.8	1.05	0.647
Globulin (g/L)	48.3	46.4	2.09	0.518
Albumin/Globulin	0.58	0.59	0.043	0.884
BUN^a^ (mmol/L)	9.13	7.67	0.689	0.161
Creatinine (μmol/L)	88	124	20.4	0.242
Energy substrates and enzymes				
Glucose (mmol/L)	5.13	4.75	0.221	0.246
Triglyceride (μmol/L)	0.223	0.180	0.0225	0.222
Cholesterol (mmol/L)	1.29	1.51	0.141	0.272
HDL^b^ (mmol/L)	0.471	0.367	0.0564	0.216
LDL^c^ (mmol/L)	0.573	0.609	0.0652	0.705
α-amylase (U/L)	21.7	18.7	4.09	0.612
Lipase (U/L)	61.4	40.6	14.42	0.327
Liver function				
ALT^d^ (U/L)	13.9	16.4	1.83	0.350
AST^e^ (U/L)	109	121	10.4	0.435
ALP^f^ (U/L)	475	358	51.3	0.133

a *BUN, blood urea nitrogen*.

b *HDL, high density lipoprotein cholesterol*.

c *LDL, low density lipoprotein cholesterol*.

d *ALT, alanine transaminase*.

e *AST, aspartate transaminase*.

f *ALP, alkaline phosphatase*.

### Feed Intake Behaviour

**Figure 2:**
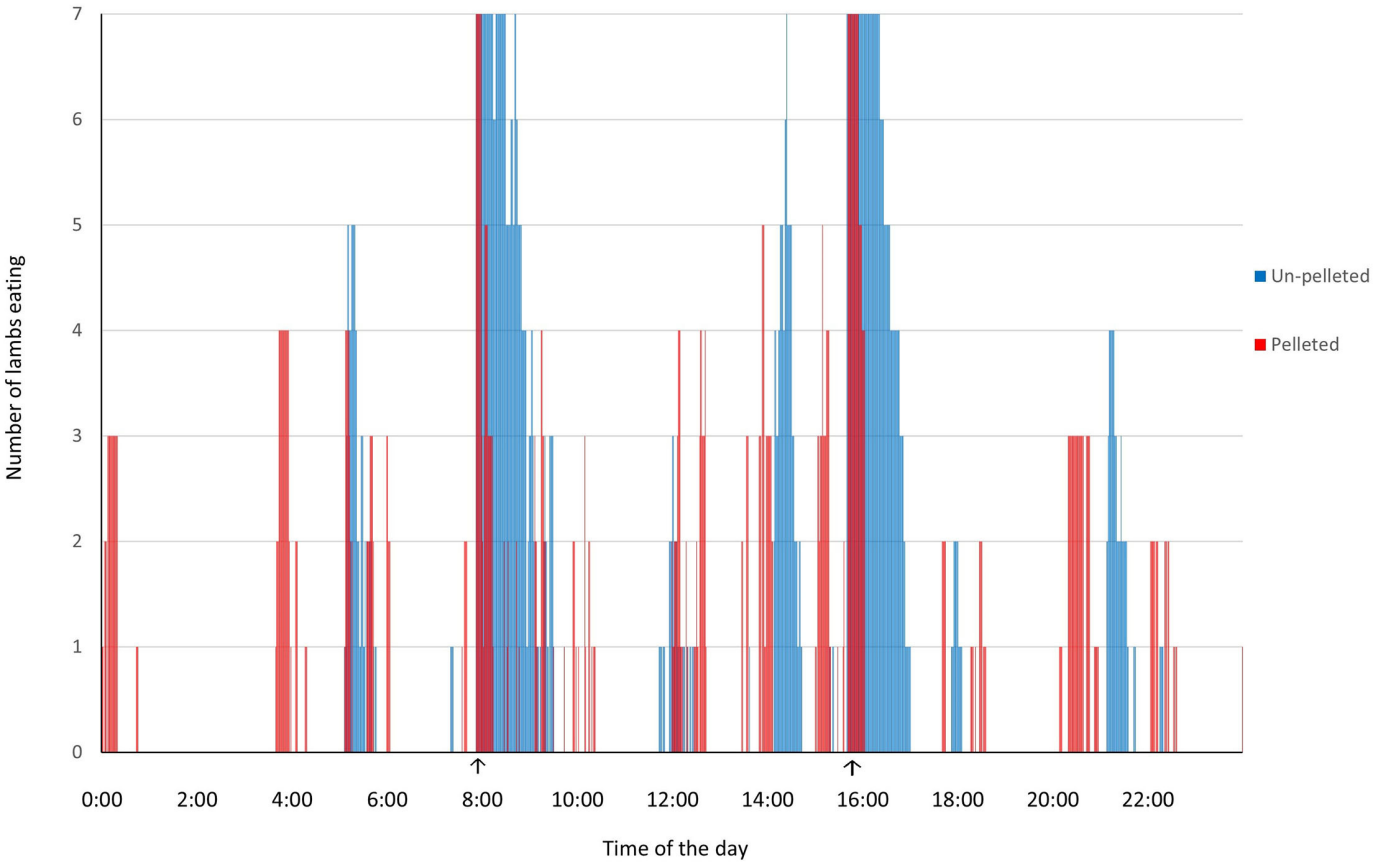
Effect of feeding pelleted TMR on feed intake activity of fattening lambs throughout the day (arrows indicate feeding times).

In Exp 1, lambs consuming pelleted feed spent 115 min/d for feed intake, which was 85 min/d shorter (*P* < 0.001) than the feed intake time (200 min/d) of lambs consuming un-pelleted feed. The time distribution of feed intake was concentrated to a relatively short period of time after feeding for pelleted feed and more evenly distributed over 24 h for lambs fed un-pelleted feed (Figure 2).

### Slaughter Performance and Meat Quality

In Exp 1, liveweight before slaughter and hot carcass weight after slaughter were 5.0 kg (*P* = 0.015) and 3.2 kg (*P* = 0.009) higher, for lambs fed pelleted feed (Table 7). In Exp 2, the differences in body and hot carcass weights were not statistically significant between the two groups, although lambs receiving pelleted feed had numerically higher body weight (+1.7 kg) and hot carcass weight (+1.1 kg). Dressing percentage was not affected by the physical form of the feed in both experiments. Eye muscle area, meat colour, marbling, brightness, pH_36_, shear force, and water holding capacity were similar between the two dietary treatments with the exception of pH_36_ being slightly higher (*P* = 0.067) by 0.08 units for lambs fed pellets than those fed un-pelleted feed in Exp 2. In Exp 1, no lambs receiving pellets and two lambs receiving the un-pelleted diet had visible lung lesions, while in Exp 2, two lambs receiving pellets and three lambs receiving the un-pelleted diet had visible lung lesions.

**Table 7:** Effects of feeding pelleted vs. un-pelleted feed on slaughter performance of fattening lambs and meat quality of the *longissimus dorsi* muscle (*n* = 6 per treatment).

		**Diet**			
**Exp**	**Item**	**Pelleted**	**Un-pelleted**	**SEM**	***P-*value**
1	Liveweight before slaughter (kg)	43.8	38.8	1.20	0.015
	Hot carcass weight (kg)	20.1	16.9	0.69	0.009
	Dressing percentage (%)	45.8	43.7	0.94	0.146
	Eye muscle area (mm^2^)	717	666	34.3	0.311
	Meat colour^a^	4.6	4.3	0.29	0.437
	Marbling^a^	1.3	1.4	0.13	0.664
	Brightness^a^	71	86	9	0.248
	${\text{pH}}_{36^{\text{a}}}$	5.98	5.90	0.027	0.067
	Shear force (kgf/cm^2^)^a^	2.06	1.46	0.247	0.118
	Water holding capacity (%)^a^	6.16	7.33	0.636	0.224
2	Liveweight before slaughter (kg)	49.9	48.2	1.12	0.302
	Hot carcass weight (kg)	22.8	21.7	0.60	0.213
	Dressing percentage (%)	45.7	45.1	0.50	0.422
	Eye muscle area (mm^2^)	843	809	51.7	0.652
	Meat colour	5.3	5	0.28	0.426
	Marbling	1.25	1.25	0.144	1.000
	Brightness	89	87	0.8	0.237
	pH_36_	5.92	5.93	0.034	0.907
	Shear force (kgf/cm^2^)	1.98	1.64	0.278	0.410
	Water holding capacity (%)	6.9	6.0	0.93	0.485

a *Meat colour, brightness, marbling, pH value, shear force, and water-holding capacity were measured after hanging for 36 h at 4°C. Meat colour and marbling were scored using the US NPPC meat quality scoring card. Meat brightness was measured using the OPTO-STAR carcass colour monitor*.

### Economic Benefits

Profit per animal was higher from lambs fed pellets than those fed un-pelleted feed (Table 8; *P* ≤ 0.04).

**Table 8:** Effects of feeding pelleted vs. un-pelleted feed to fattening lambs on economic profit and feed cost per kg of liveweight gain.

		**Diet**			
**Exp**	**Item (unit: US dollars)**	**Pelleted**	**Un-pelleted**	**SEM**	***P*-value**
1	Profit per lamb	22.62	6.77	1.969	<0.001
	Feed cost per kg of liveweight gain^a^	1.94	2.27		
2	Profit per lamb	8.77	6.00	0.554	0.004
	Feed cost per kg of liveweight gain	3.15	3.62		

a *The cost of pelleting was included in the feed cost*.

## Discussion

### Pelleting on Growth Performance and Apparent Total Tract Digestibility of Fattening Lambs

In the present study, lambs fed TMR pellets had 37–70 g/d higher ADG than those fed un-pelleted TMR. These results are consistent with Coufal-Majewski et al. (44), Zhong et al. (11), and Zhang et al. (13). In these reports, the increase in ADG in response to feeding TMR pellets was 60 (44), 47 (11), and 76 g/d (13) compared to un-pelleted TMR. Improved growth performance was also demonstrated by higher carcass weight in Exp 1 of this study and Zhang et al. (13).

Growth performance is foremost impacted by total feed intake and the quantity of nutrients animals are able to utilise per unit of ingested feed. Lambs ate more when the feed was pelleted in this study. This finding is in agreement with earlier (45) and more recent (11, 13, 46) reports. The increase in DM intake is mainly due to the reduction in rumen fill in response to pellets, which allows greater feed intake to reach satiety. Increased feed intake can at least partly explain improved growth performance. As it was the case in this study, pelleting does not always improve feed conversion efficiency. This is consistent with Coufal-Majewski et al. (44), Zhong et al. (11), and Zhang et al. (13). In our study, pelleting resulted in a slight decrease in DM digestibility, but the digestibility of all other measured nutrients did not change. No difference in digestibility was also reported by Zhang et al. (13) and Coufal-Majewski et al. (44). In contrast, Zhong et al. (11) found the digestibility of CP, ADFom, ether extract, and starch slightly increased due to pelleting and the digestibility of DM and aNDFom remained unchanged. Karimizadeh et al. (46) reported an increase in digestibility of DM and ADF with pelleting. Feed pelleting affects the digestibility of nutrients since processing conditions such as temperature, duration, and water content have effects on nutrient degradation (47–49). Different pelleting conditions among studies may be one of the reasons for the discrepancy in digestibility response. Differences in lamb breed, age, and sex across studies could be another reason. However, the difference in digestibility is small, and increased feed intake may be the main reason for improved growth performance.

### Pelleting on Rumen Fermentation and Microbial Community

Rumen pH is an important fermentation parameter and affected by a range of factors, including feed processing (50). In the present study, rumen pH decreased due to pelleting, especially 3 h after the morning feeding. Samples taken from the rumen, reticulum, and omasum after slaughter also had lower pH values in lambs fed pellets compared to those fed un-pelleted feed. The lower pH value in lambs fed TMR pellets may be related to faster eating observed in this study and by Karimizadeh et al. (46) and higher feed intake (11, 13, 46). The more feed ingested in a short time provides rumen microbes more substrates to ferment. This is evident by higher concentrations of ammonia and total SCFA observed in this study and Zhong et al. (11). Lower rumen pH and higher total SCFA were also observed in cattle fed pelleted feed (51). Even though, the concentrations of SCFAs in the study by Zhang et al. (13) were higher in lambs fed pelleted compared to un-pelleted TMR, rumen pH was not affected. However, Zhang et al. (13) collected rumen samples after slaughter from lambs that were fasted for 12 h, which may have impacted the outcome of the rumen pH sampling. It is worth to note that in this study and other studies comparing the effect of pelleting TMR for lambs (11, 13, 46) rumen pH was within range required for normal physiological function and did not induce acidosis. In a study conducted with dairy cows, rumen pH was within the normal range, and no clinical signs of rumen acidosis were observed, but the authors raised caution concerning the potential risk of acidosis due to a 3.5% drop in milk yield and a 8.2% decrease in milk fat to milk protein ratio in response to feeding pelleted TMR (7). All studies cited in this section lasted for <2 months. Consequently, long-term and dynamic measurements of rumen pH are needed in future studies to fully understand in how far rumen pH is affected by feeding pelleted TMR.

The three most abundant bacterial phyla were Bacteroidetes, Firmicutes, and Proteobacteria, accounting for 95.8% of the total bacterial population in this study. This profile of bacteria community is consistent with other studies feeding diets with similar chemical composition to lambs (12) and the two most abundant phyla (Bacteroidetes and Firmicutes) are the same as reported by Zhang et al. (13). The most abundant bacteria at genus level were *Prevotella 1, Prevotella 7, Rikenellaceae RC9 gut group, Succinivibrio, Ruminococcus 2, Succinivibrionaceae UCG-001, Selenomonas 1*, and *Lachnospiraceae NK3A20 group*. These genera were not affected by the physical forms in this study. The stability of the most abundant bacteria may reflect the presence of the core microbiome (52).

Among the studies comparing pelleted and un-pelleted feed in sheep, only Zhang et al. (13) analysed the rumen microbial composition. Zhang et al. (13) found that feeding pelleted TMR resulted in a shift of rumen microbiota composition in fattening lambs. These findings are different from our results. However, Zhang et al. (13) only sampled 5 lambs, while 15 lambs were sampled in our study. Our experience suggests that at least 7–8 animals per treatment are needed to obtain reliable rumen microbial comparison results, this is in line with other studies [e.g., Martinez-Fernandez et al. (53)]. Large animal-to-animal variation in rumen microbial composition might produce false results when the number of samples is limited (54). Further studies are warranted to draw a solid conclusion regarding whether or not pelleting affects rumen microbes.

### Pelleting on Serum Parameters

Serum parameters are frequently measured as indicators of the nutritional status, physiological state, and immune function of animals. Differences of these parameters in response to pelleting did not reach statistically significant levels, but agreed with the results reported in the literature. Blood urea nitrogen is an indicator of nitrogen status in ruminant body and is affected by the dietary intake and degradation of crude protein (55). Blood urea nitrogen is positively associated with ammonia concentration in the rumen (56, 57). The increased ammonia concentration in the rumen with feed pelleting in this study, which agrees with the findings of Karimizadeh et al. (46), Zhong et al. (11) and Zhang et al. (13), led to a numerically higher blood urea nitrogen concentration in lambs fed the pelleted TMR. The concentrations of TP, ALB, and GLB in blood reflect the level of body immunity (58). In the present study, serum TP and GLB concentrations were numerically higher in lambs fed pellets, which is consistent with the results of Zhong et al. (11), suggesting the improvement of animal health. Lipoproteins in blood have a function of lipid transportation and are considered to be related to animal health. Zhong et al. (11) found an increase in HDC and a decrease in LDC in plasma of lambs fed TMR pellets. In contrast, we did not find these differences in serum. Further studies are warranted to clarify the effects of pelleting on lipoproteins.

### Pelleting on Slaughter Performance and Meat Quality

Lambs fed pelleted feed had higher body weight and hot carcass weight than those fed un-pelleted feed. This may have resulted from the higher feed intake leading to better growth performance with pellet-fed lambs. Dressing percentages were similar between the two feed physical forms in the study. While ADG is negatively associated with dressing percentage, large variation in dressing percentage attribute to other factors (59, 60). Although ADG was higher for lambs fed pelleted feed in our study, this difference did not appear to have a significant effect on dressing percentage. Large eye muscle area is associated with increased lean meat yield in lambs (61). Pelleting did not result in a difference in eye muscle area in this study. When different forages were used to feed lambs, eye muscle area remained unchanged although growth performance was largely affected (62). It seems that feed composition or physical form has less effect than genetics on eye muscle area (61).

Meat colour is a main factor influencing the purchasing behaviour of consumers (63). There were no significant differences in meat colour and brightness between the two treatments in both experiments, indicating that the feed physical form had no significant effect on meat colour. Marbling is one of the important reference indexes of meat quality, and also an important factor to determine the price of lamb meat (63). In this study, the effect of different feed forms on marbling was not significant, and the score of marbling was low. This may be due to the fact that fattening lambs have not reached maturity at slaughter, and there was still potential for further growth and aggregation of fat. The energy consumed was mainly for growth, and there was no excess energy for fat deposition. Other meat quality indicators were also similar between the two feed physical forms. These results suggest that feed pelleting does not affect meat quality.

### Pelleting on the Profitability of Lamb Fattening

Although feed pelleting increases production cost (64), animal growth performance was improved in lambs fed pelleted TMR in the present and other studies (11, 13, 46). This leads to a low ratio of liveweight gain to feed and an increase in profit. The cost for per kg of liveweight gain decreased by US$0.33 to 0.47 in this study. Improved growth performance would also shorten the time needed until lambs reach market weight. In addition, feeding pelleted feed would reduce feed waste and increase labour efficiency, which would further improve profitability.

## Conclusions

Pelleting of a total mixed ration improves the average daily feed intake and the average daily gain of fattening lambs. Feed pelleting does not affect apparent total tract nutrient digestibility but reduces feeding time and feed wastage. Feeding pelleted feed increases the total amount of short-chain fatty acids and decreases the pH value in the rumen. Serum enzymes and metabolites are not affected by the physical form of the feed. Feeding pelleted total mixed rations can shorten the fattening time and has no negative impact on meat quality. The profitability of lamb fattening improves in response to pelleting. This study supports that pelleting of a total mixed ration is a feasible technique for lamb fattening.

## Data Availability Statement

The datasets presented in this study can be found in online repositories. The name of the repository and accession number can be found at: NCBI BioProject, accession no: PRJNA681973. http://www.ncbi.nlm.nih.gov/bioproject/681973.

## Ethics Statement

The animal study was reviewed and approved by Animal Ethics and Welfare Committee of Jilin Agricultural Science and Technology University (Approval number 2018003).

## Author Contributions

XS and PY conceived and planned the study. XS acquired funding and supervised all research. BL, QH, GZ, YH, WT, RL, CL, JL, CW, BS, and XS collected data. XS and BL analysed and interpreted data, prepared the tables and prepared the figures, and wrote and/or revised the manuscript. All the authors approved the final version of the manuscript.

## Conflict of Interest

PY was employed by Portal Agri-Industries Co., Ltd. The remaining authors declare that the research was conducted in the absence of any commercial or financial relationships that could be construed as a potential conflict of interest.
