# Supplementary material for: Pelleting of a Total Mixed Ration Affects Growth Performance of Fattening Lambs
Source: Front Vet Sci. 2021 Feb 12;8:629016. doi: 10.3389/fvets.2021.629016 (PMC7928353; doi:10.3389/fvets.2021.629016)
Supplement: Supplementary file 1 [file Table_1.DOCX]

**Supplementary Table 1.** Effects of feeding pelleted vs. un-pelleted feed on pH value of lamb digestive tract chyme in Exp 2 (*n* = 6 per treatment).

|  | **Diet** | |  |  |
| --- | --- | --- | --- | --- |
| **Digestive tract** | **Pelleted** | **Un-pelleted** | **SEM** | ***P* value** |
| Rumen | 5.99 | 6.26 | 0.081 | 0.041 |
| Reticulum | 6.12 | 6.54 | 0.099 | 0.012 |
| Omasum | 6.05 | 6.43 | 0.114 | 0.042 |
| Obomasum | 3.85 | 3.39 | 0.238 | 0.206 |
| Caecum | 6.48 | 6.53 | 0.036 | 0.352 |
