# Supplementary material for: Pelleting of a Total Mixed Ration Affects Growth Performance of Fattening Lambs
Source: Front Vet Sci. 2021 Feb 12;8:629016. doi: 10.3389/fvets.2021.629016 (PMC7928353; doi:10.3389/fvets.2021.629016)
Supplement: Supplementary file 2 [file Table_2.DOCX]

**Supplementary Table 2.** Effects of feeding pelleted vs. un-pelleted feed on the relative abundance (%) of ruminal bacteria at the level of phylum in fattening lambs, samples collected before the morning feeding (n = 15 per treatment).

|  | **Diet** | |  |  |
| --- | --- | --- | --- | --- |
| **Phylum** | **Pelleted** | **Un-pelleted** | **SEM** | ***P* value** |
| Bacteroidetes | 59.3 | 58.0 | 4.34 | 0.826 |
| Firmicutes | 27.7 | 31.9 | 3.43 | 0.386 |
| Proteobacteria | 7.9 | 6.7 | 2.98 | 0.78 |
| Unclassified | 2.3 | 0.3 | 1.26 | 0.285 |
| Spirochaetes | 0.8 | 1.4 | 0.42 | 0.315 |
| Actinobacteria | 0.5 | 0.5 | 0.13 | 0.882 |
| Fibrobacteres | 0.6 | 0.3 | 0.13 | 0.076 |
| Cyanobacteria | 0.2 | 0.3 | 0.17 | 0.613 |
| Patescibacteria | 0.3 | 0.2 | 0.07 | 0.103 |
| Tenericutes | 0.2 | 0.1 | 0.07 | 0.347 |
| Synergistetes | 0.2 | 0.1 | 0.07 | 0.314 |
| Elusimicrobia | 0.0 | 0.2 | 0.09 | 0.219 |
